# Cerebral Autoregulation Real-Time Monitoring

**DOI:** 10.1371/journal.pone.0161907

**Published:** 2016-08-29

**Authors:** Adi Tsalach, Eliahu Ratner, Stas Lokshin, Zmira Silman, Ilan Breskin, Nahum Budin, Moshe Kamar

**Affiliations:** Ornim Medical Ltd, Kfar Saba, Israel; Hopital Robert Debre, FRANCE

## Abstract

Cerebral autoregulation is a mechanism which maintains constant cerebral blood flow (CBF) despite changes in mean arterial pressure (MAP). Assessing whether this mechanism is intact or impaired and determining its boundaries is important in many clinical settings, where primary or secondary injuries to the brain may occur. Herein we describe the development of a new ultrasound tagged near infra red light monitor which tracks CBF trends, in parallel, it continuously measures blood pressure and correlates them to produce a real time autoregulation index. Its performance is validated in both *in-vitro* experiment and a pre-clinical case study. Results suggest that using such a tool, autoregulation boundaries as well as its impairment or functioning can be identified and assessed. It may therefore assist in individualized MAP management to ensure adequate organ perfusion and reduce the risk of postoperative complications, and might play an important role in patient care.

## Introduction

Cerebral autoregulation (AR) refers to the intrinsic ability of the brain’s vasculature to react to changes in arterial blood pressure (BP), in order to maintain stable cerebral blood flow (CBF)[1–3]. Lassen et al.[1] have reported an AR curve which was set as standard, comprising of a CBF plateau with lower and upper limits in mean arterial pressure (MAP) values of 50 to 150mmHg. However, these limits are subject to inter-patient variations[4], and alterations controlled by factors that change CBF and its vasorectivity, such as the sympathetic activity or the vascular rennin-angiotensin system[5]. In chronic hypertension for example, the limits of AR are shifted toward higher blood pressures[6]. AR may be further compromised by certain disease states of the brain, ranging from impairment to non-function, leaving the brain unprotected against potentially harmful effects of BP changes. In severe head injury or acute ischemic stroke, autoregulation may be impaired or even lost[7, 8]. Thus, assessing whether this mechanism is intact or impaired, and determining the boundaries of the aforementioned plateau, is important in many clinical settings, where primary or secondary injuries to the brain may occur due to hypo or hyperperfusion. However, until today, no real time monitor for AR functioning exists.

Over the years several methods were introduced to examine AR, all using surrogates indices, such as PRx (using intra cranial pressure (ICP))[9–11], Mx (using CBF Velocity (CBFV) measured by Transcranial Doppler (TCD))[12–14], COx (using cerebral oximetry measured by Near Infrared Spectroscopy (NIRS))[14, 15], and others. Though many additional parameters may be associated with cerebral autoregulation, its basic mechanism is usually described by either the static or dynamic relationship between MAP and CBF[1, 2, 16, 17]. Various analysis techniques were developed to assess and quantify the association between these two parameters in either time or frequency domains[18–20]. Few examples include: assessment of the rate of recovery of CBF in response to a MAP challenge[2], calculation of an autoregulation index based on mathematical models[21], and computation of a transfer function with evaluation of its properties[22, 23].

A common analysis method incorporates calculation of the correlation between CBF to cerebral perfusion pressure[24] or MAP[12]. In this method, a moving linear correlation coefficient is calculated for a certain time window and evaluated over time. Correlation coefficients approaching 1 are associated with loss of autoregulation as CBF is dependent on MAP changes (i.e. “pressure-passive”), while lower values, approaching zero, are related to cases in which autoregulation is intact. This method was used to continuously monitor autoregulation in numerous studies and various disease\clinical states. For these studies, a correlation coefficient threshold value of 0.4–0.45 was established to distinguish between intact and impaired autoregulation[13, 14, 25]. The value is dependent upon the correlated signals (CBF surrogate), calculation procedure, etc. This correlation technique, however, was mostly applied in post processing rather than providing a real time AR indication, as no device incorporating both measurements is present.

Ornim’s acousto-optic based device (c-FLOW and its predecessor the CerOx) is a noninvasive, continuous CBF monitor[26–29]. The ability to perform offline calculations of the AR state using its cerebral flow index (CFI) and an independent MAP measurement was demonstrated in previous studies[28, 29]. Herein we describe the development of a new AR monitor which correlates a direct CBF signal with concurrent MAP measurement to produce a real time autoregulation index (ARI).

## Methods

The c-FLOW is an ultrasound tagged light based device, which utilizes near infrared laser light (808nm) modulated by low power 1MHz ultrasound, to perform continuous real time blood flow monitoring in the tissue microcirculation. Being a non-invasive device, its sensors are usually placed on the patient’s forehead and provide local microcirculation CBF monitoring in approximately 1cm^3^ volume, located 2cm deep underneath them. Due to the use of ultrasound, placement of the sensors on every other location but the forehead requires pre-shaving of the region of interest. The c-FLOW provides a Cerebral Flow Index (CFI) which describes changes in cerebral blood flow in arbitrary units between 0–100, where 0 represents no flow. It is also capable of setting a baseline flow value and presenting the percent flow change from this baseline. Each CFI value represents a moving average of the last 30 seconds of ultrasound tagged near infra red (UT-NIR) signal and is updated every 2–3 seconds. The specifics on how it operates and eliminates the effect of superficial flow are detailed elsewhere[30].

Due to the use of ultrasound and light, the c-FLOW has several inherent limitations. As every other NIR based device, its measurement depth is confined to the cortex only because of light absorption in tissues. In addition, since it includes ultrasound, good coupling between the sensors to the skin is obtained by US gel which has to be renewed every few hours.

To enable autoregulation monitoring, a modified version of the c-FLOW that includes a BP unit was developed, termed c-FLOW-AR. This unit connects to a standard invasive blood pressure (IBP) sensor, and enables tracking MAP concurrently with CFI and display both trends synchronically. The real time combination of these two parameters enables the calculation of a correlation index (termed ARI), which reflects the interrelationship between changes of MAP to those of CFI. ARI values range between 0 to 100, where 0 represents a condition of no correlation between MAP and CFI changes, while 100 represents a perfect one. Accordingly, when cerebral autoregulation is intact, a change in MAP is not followed by a corresponding change of CBF, as the brain autoregulates. In this case, ARI will get lower values (approaching zero). If autoregulation is impaired, it is expected that changes in MAP will cause corresponding changes in CBF and ARI should get higher values.

ARI is calculated only for time intervals in which marked MAP changes exist. Identification of notable MAP changes is obtained by a Relevance Vector Machine (RVM) linear classifier based on the trend slope and derivatives. For all other time intervals, in which MAP is approximately constant, no ARI is calculated. The ARI is therefore the reflection of the autoregulation status, once changes in marked MAP occur.

The ARI value is obtained by calculating the cross correlation maxima in a predetermined time interval (5 minutes). In this interval, the correlation values between CFI and MAP are calculated for all time shifts. They are then multiplied by a time-shift dependent weight function, in which the weight decays as time shift increases, and the maximum obtained value is selected. In this way, the ARI compensates for the intrinsic physiological time delays between changes in systemic blood flow and pressure to those of the brain.

### *In-vitro* experiments

CBF was modeled using a previously described acousto-optic phantom which mimics blood flow in tissues[30, 31]. The phantom is made of Dermasol (CA medical innovations) which is a synthetic polymer matrix soaked with oil. Titanium Dioxide (TiO_2_) particles (0.1% by weight) were added as light scattering agents. The optical and acoustic properties of the phantom are similar to those of tissue, as detailed in Table 1.

**Table 1:** optical and acoustic properties of the phantom and the tissue[32, 33].

Property	Tissue–muscle/brain	Phantom
Light Effective decay coefficient	2.17/2.12 cm^-1^	2.2 ±0.2cm^-1^
Sound Velocity	1.5 ·10^5^ cm/s	1.43 ·10^5^ cm/s
Acoustic impedance	150–170 Kg/cm^2^∙s	149 Kg/cm^2^∙s

**Fig 1:**
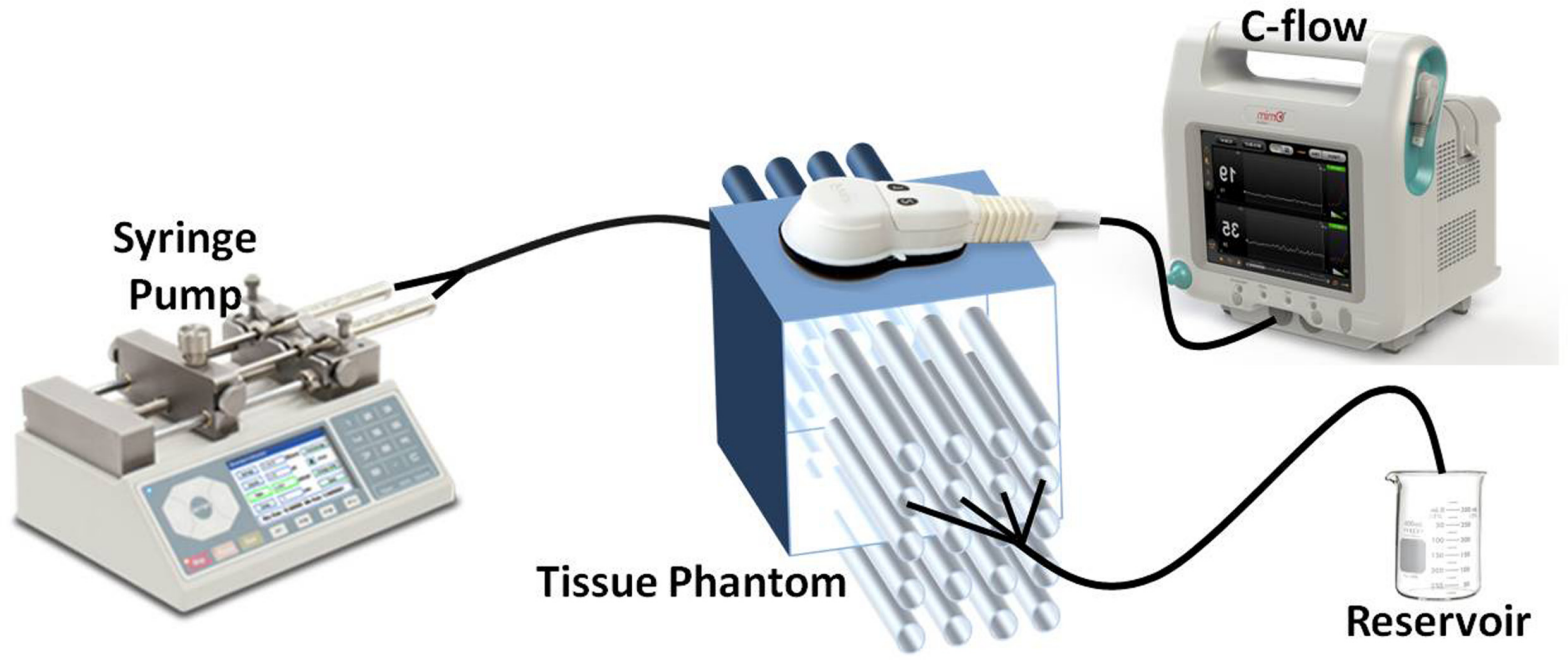
Schematic illustration of the flow modeling experimental setup. The C-FLOW’s measuring sensor is placed above the tissue phantom. Flow within the phantom’s tubes is generated using the syringe pump.

Simulating tissue microcirculation, we designed a Dermasol slab containing 20 hollow parallel channels with an outer diameter of 1mm through which scattering fluid (similar to blood) can flow. A schematic diagram of the experimental set-up is presented in Fig 1.

Flow within the phantom’s tubes was generated using a calibrated computer-controlled syringe pump (Chemix, Model Infusion 200) and measured using the c-FLOW-AR’s (Ornim Medical, Israel) sensor which was placed above. Though flow within cerebral microcirculation is multidirectional, flow within the phantom’s channels was linear. As the c-FLOW is insensitive to flow direction, this simplified model was sufficient to demonstrate the device’s capabilities.

**Fig 2:**
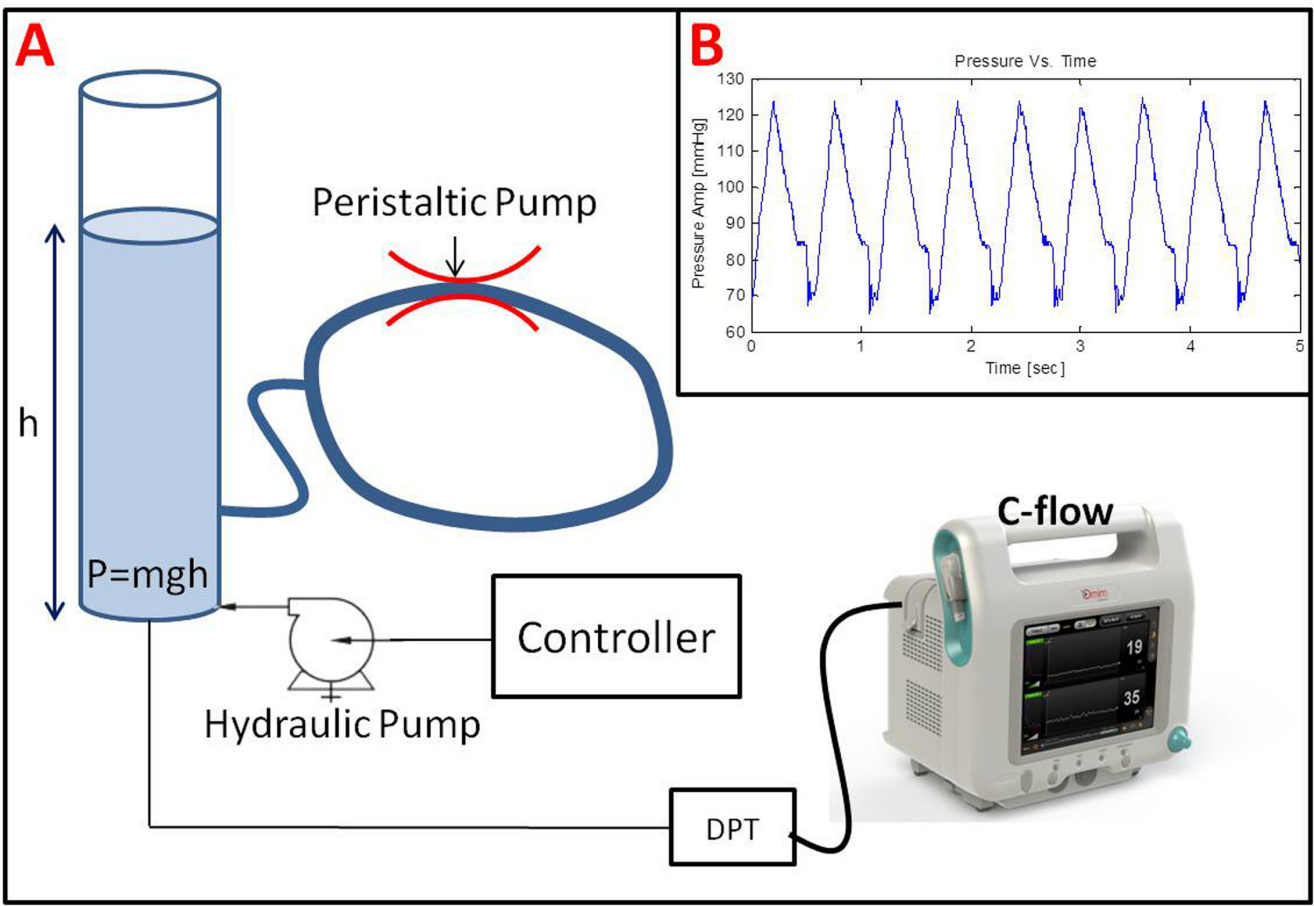
(A) Schematic illustration of the pressure modeling experimental setup comprising of a water column connected to a computer controlled hydraulic pump and a peristaltic pump. Pressure was measured using a standard disposable pressure transducer (DPT) connected directly to the C-FLOW-AR. (B) An example of a pressure wave generated by the hydraulic pressure system designed to mimic blood pressure. All pressure properties, such as average pressure (MAP), oscillations magnitude (Systolic-Diastolic pressures), and frequency (HR), are controlled and can be predetrmined.

BP was modeled using a hydraulic pressure system that was able to generate periodical hydrostatic pressure, similarly to blood pressure. The following experimental setup was utilized (Fig 2A).

Constant static pressure was created by a water column of a predetermined height (h), as illustrated on the left of Fig 2(A). The height (h) relative to the pressure sensor was digitally changed using a computer controlled hydraulic pump and a pressure controller, thus creating different static pressure levels, corresponding to different MAP values. To create pressure oscillations around the static pressure (pulsatile pressure), the water column was hydraulically connected to a ring-like tube filled with water. A peristaltic pump (MasterFlex L/S easy-load II, model 77200–62) connected to that tube was used to create pressure variations over time. The water column was hydraulically connected to a standard disposable pressure transducer (DPT), similar to the one used in arterial lines (Art-Line^TM^ Biometrix, Israel), and directly connected to the c-FLOW-AR. Example for a pressure wave generated by the described hydraulic pressure system, measured by the c-FLOW-AR is illustrated in Fig 2(B).

A designated LabView® program was used to control both the flow and the pressure systems synchronically. To examine the ARI performance in different AR conditions, the following protocol was applied. Each of the two cFLOW-AR’s sensors was placed on a different acousto-optic flow phantom (depicted in Fig 1) to enable the implementation of different flow protocols to each of the sensors. Average pressure (MAP) was raised and lowered with a notable amplitude change (from 100mmHg to 180mmHg). Flow in phantom number 1 (measured by sensor number 1) was changed in accordance with MAP changes (modeling pressure passive condition), while flow in phantom number 2 (measured by sensor number 2) was kept constant (modeling intact AR). In such a way, both cases of impaired and intact AR were simulated simultaneously (with sensors 1 & 2 respectively).

Mean pressure, flow index and the correlation (ARI) between them were real-time displayed on the C-FLOW-AR’s screen and automatically saved to the device hard drive for post processing purposes. The coefficient of variance was used to estimate the homogeneity of CFI as measured by the two c-FLOW-AR’s sensors and validate the applied flow protocols. ARI values measured for the two different flow protocols were compared using independent t-test. Significance level was defined as α = 0.05.

### Preclinical case study

A case study of real-time assessment of a swine autoregulatory state is presented. The procedures were approved by “Asaf Harofe” medical center Institutional Animal Care and Use Committee (IACUC) and conducted in strict accordance with the guideline for animal care and use established by the IACUC.

#### Animal preparation

A female piglet (Sus domestica, 2–3 months old), weighing 25.6Kg was anaesthetized with an initial bolus of IM Ketamine, 1.5mg/Kg and Xylazine 2mg/Kg. After induction, the animal was intubated and mechanically ventilated, keeping SaO_2_ above 93% and ETCO_2_ at 35–40mmHg. Anesthesia and analgesia was maintained using IV Propofol 0.02–0.1 mg/Kg/min and Fentanyl 0.015 mcg/Kg/min. An arterial line was inserted to the carotid artery for BP monitoring. To avoid hair and enable both sensors adhesion to the skin and good US coupling, the animal’s head was shaved (~10x10cm area) and cleaned with alcohol solution. The skin remained intact with no visible scratches or wounds.

#### Monitoring

MAP was measured using c-FLOW-AR monitor (Ornim Medical, Israel) via arterial line introduced to the carotid artery. CFI was monitored with a non-invasive sensor (5x2.5x1.5cm) placed on the skin surface of animal’s forehead connected to the c-FLOW-AR. Correlation Index between MAP and CFI (ARI) was calculated in real time and presented on the monitor.

Heart rate and ventilation parameters (respiratory rate, end tidal CO_2_, arterial saturation) were continuously monitored using non invasive pulse oximetry and capnography (Novametrix, USA).

#### Experimental procedure

Cardiac preload, and therefore cardiac output, was optimized using fluid boluses of 5–10 ml/kg, while monitoring BP and PaO_2_. After MAP stabilization, experiment began. Baseline CFI was recorded for a period of 15–30 minutes prior to starting each manipulation.

To create MAP variations, BP was pharmacologically manipulated. Intravenous (IV) Phenylephrine (50 mcg/ml) was used to increase MAP. Incremental dosages were infused, starting at 2.5 ml/hr and increasing in 2.5 ml/hr every 7–10 minutes, until MAP doubled from baseline. Once targeted MAP was reached, Phenylephrine injection was stopped for 30–60 minutes and a new baseline was acquired.

IV Nitropruside (2 mg/ml) was used to decrease BP. Incremental dosages, starting at 2 ml/hr and increasing in 2 ml/hr were infused every 7–10 minutes, until MAP dropped by 50% or reached 40 mmHg. Once targeted MAP was reached, Nitroprusside stopped for 30–60 minutes allowing the animal to stabilize in a new baseline.

#### Data Collection

MAP, CFI, and the calculated correlation index (ARI) were digitally saved to the c-FLOW-AR device. Other physiologic parameters were sampled using a designated LabView program and saved to an excel worksheet for analysis.

#### Data Analysis

Changes in CFI were correlated with changes in MAP throughout the monitoring period. To demonstrate the autoregulation assessment ability, CFI was plotted as a function of MAP, to illustrate the AR curve and detect its boundaries (upper and lower limits). The slope and 95% confidence interval (CI) between MAP and CFI was calculated for the two MAP regions.

ARI values were binned into groups according to their corresponding MAP values (10mmHg segments). Averaged values for each bin were presented using a columns diagram. Receiver Operating Curve (ROC) analysis was used to estimate the ARI performance in classifying MAP as under or over the limits of autoregulation.

Analyses were carried out using SPSS 23.0.01 and Matlab R2014a (8.3.0.532).

## Results

### *In-vitro* Experiments

**Fig 3:**
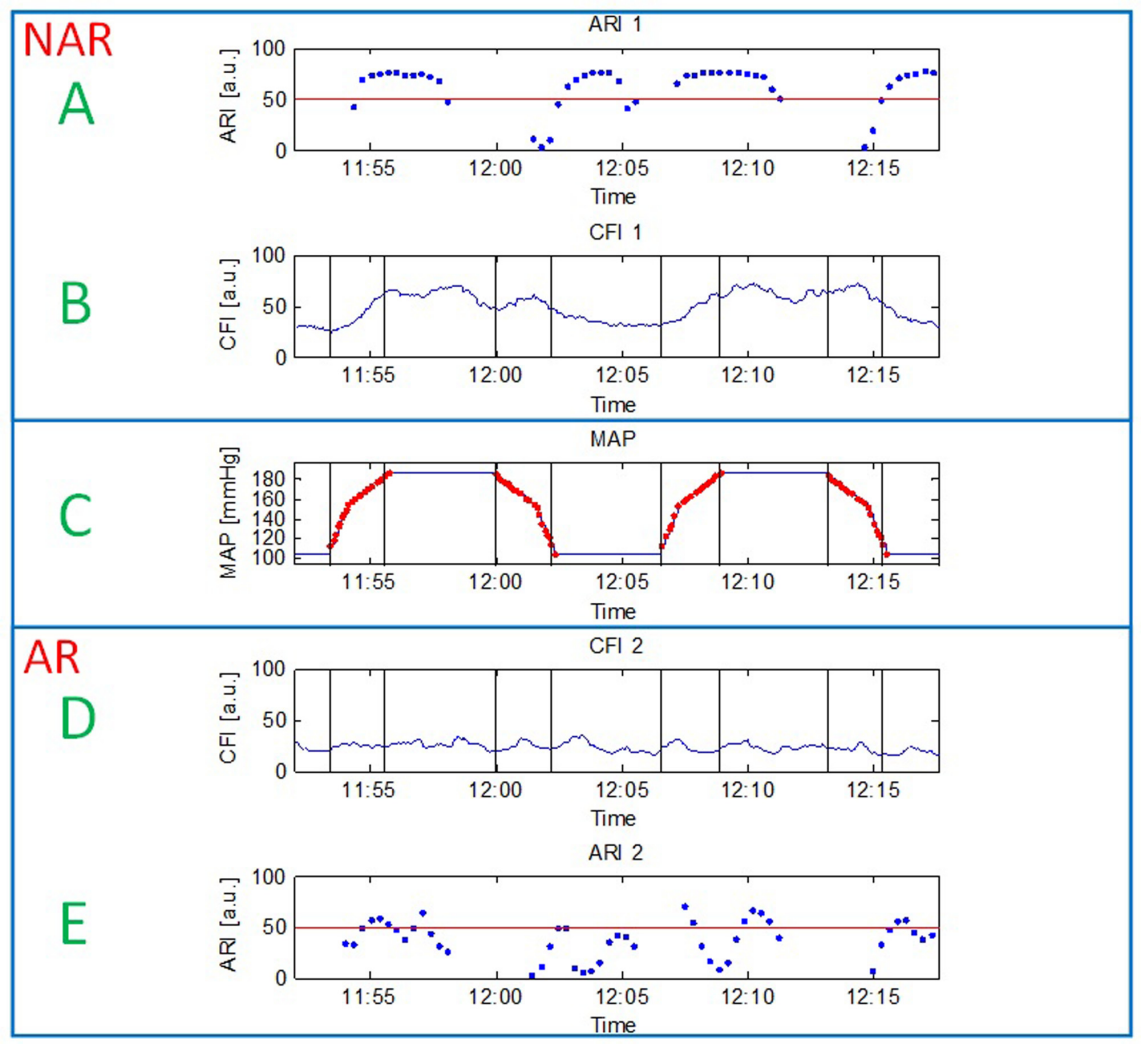
MAP (C), CFI (B,D) and ARI (A,E) data obtained in the *in-vitro* experiment. MAP (panel C) was significantly increased and decreased, followed by a similar flow protocol which was applied to sensor 1 (panel B) versus constant flow which was measured by sensor 2 (panel D). Sensor 1 which experienced a pressure passive flow protocol was used to model No AutoRegulation (NAR) state, while sensor 2 which measured constant flow was utilized to model intact AutoRegulation (AR) state. Corresponding calculated autoregulation indexes are depicted in panels A and E respectively. Delayed ARI values relative to MAP changes are due to data collection and buffering required for ARI calculation.

Laboratorial experimental setup was used to increase and decrease MAP between 100mmHg to 180mmHg (Fig 3 -Panel C). Flow measured by sensor number 1 (panel B) experienced corresponding concurrent changes (CFI = 44.93±18.18, coefficient of variance (CV) = 0.4), while flow measured by sensor 2 (Panel D) was kept constant throughout the experiment (CFI = 22.89±4.6, CV = 0.2). CV obtained for sensor 1 was twice higher than the one of sensor 2, indicating on fluctuating versus steady flow, respectively. To further validate the difference between CFI values measured by the two different sensors during the experiment, measurements were divided to periods before and after MAP increase, and the difference between groups was evaluated using t-test. Averaged CFI values obtained for the two groups with their corresponding p values are summarized in Table 2.

**Table 2:** Averaged CFI values obtained in *in-vitro* experiment.

	CFI Before MAP Increase	CFI After MAP Increase	P value
**Sensor 1**	39.80	59.61	6.63E-58
**Sensor 2**	23.35	23.84	0.21

Results show that significant difference was obtained before and after MAP increase in sensor 1 (P<0.001), while sensor 2 measured stable values (P>0.05), validating the applied manipulation. Obtained autoregulation indexes for the two disparate sensors were significantly different (ARI for sensor 1 = 61.74±21.36, ARI for sensor 2 = 37.66±17.99, Independent T-test, P<0.001). Fig 3 illustrates data over time (MAP (C), 2 CFI channels (B,D) and corresponding ARI values(A,E))) obtained in the *in-vitro* experiment.

**Fig 4:**
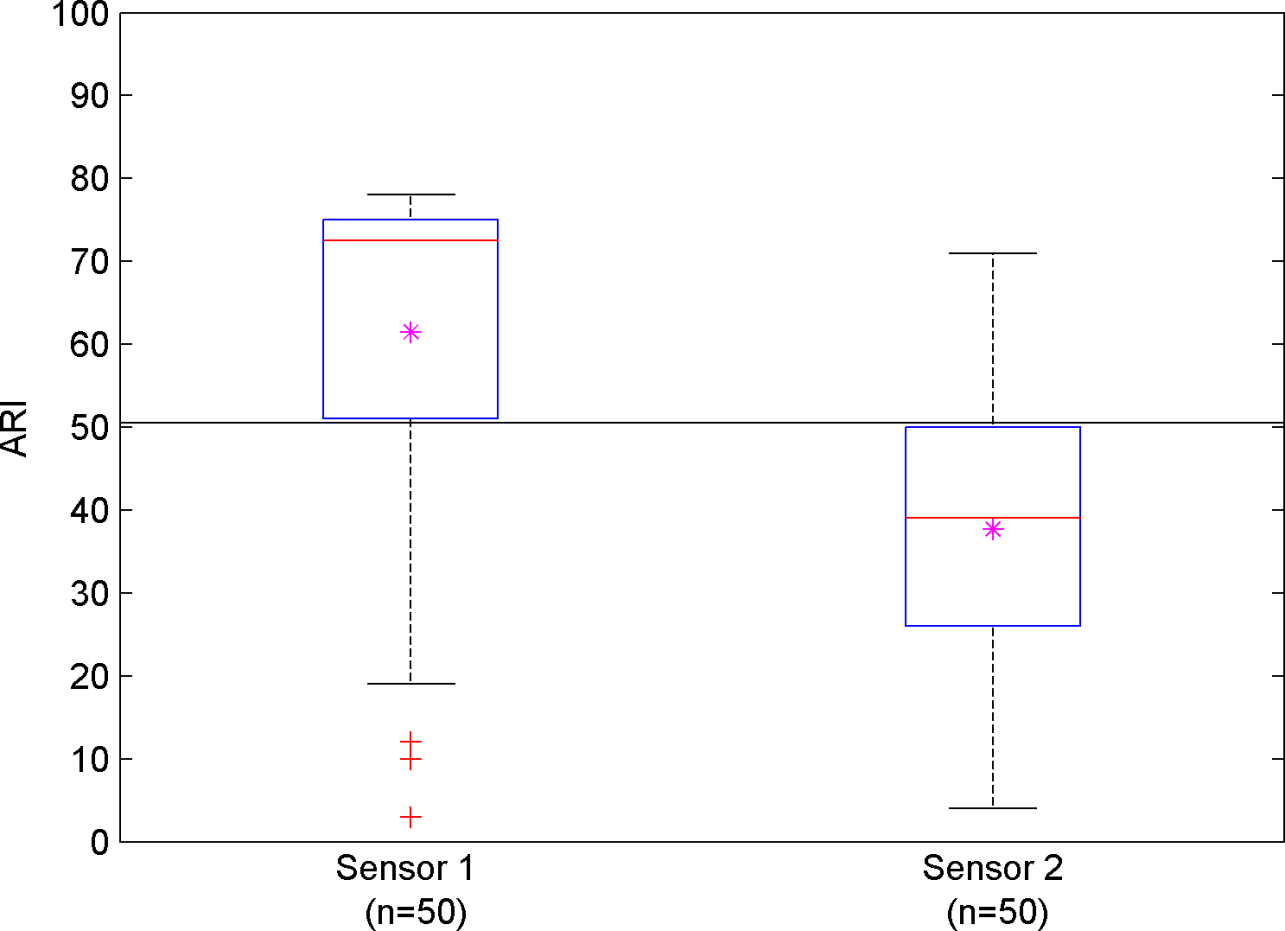
Boxplot for autoregulation index values calculated for the two to cFLOW-AR sensors. Pink astrics represent averaged ARI for each condition. A distinct separation between the conditions is apparent.

To better illustrate the distribution of the ARI values, Fig 4 demonstrates a boxplot of the ARI calculated for the two sensors, exhibiting again a distinct separation between the two groups.

### Preclinical case study

**Fig 5:**
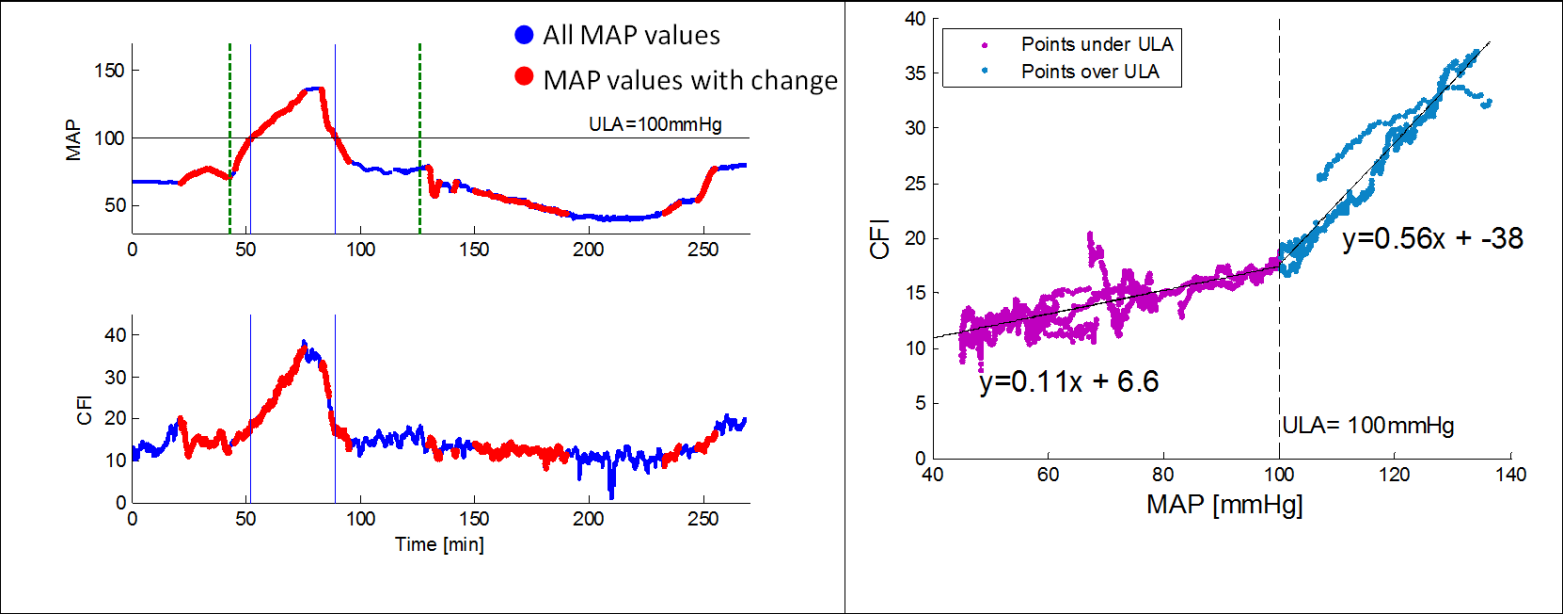
Left—MAP and CFI data over time throughout the study. MAP was increased to 140mmHg followed by a return to baseline and a decrease to 40mmHg. Dashed green lines represent initial injections of Phnylephrine and Nitroprusside respectively. Blue points represent all MAP values. Red points are associated with periods in which the algorithm identified a significant MAP change and a correlation index (ARI) can be calculated. Right—Scatter plot of CFI versus MAP revealing two distinct slopes obtained for values under of over 100mmHg. This point was defined as the upper limit of autoregulation (ULA).

MAP and CFI data were obtained, using c-FLOW-AR, for a duration of over 4 hours. Anesthesia with IV Propofol is known not to compromise AR as opposed to volatile anesthesia in high concentrations[34], therefore intact AR curve was expected to be observed. MAP was lowered to approximately 40mmHg and raised to 140mmHg using stepwise increases of drug dose. Data over time is presented in Fig 5 (left), where blue points represent all MAP values and red points are associated with periods in which the algorithm identified a significant MAP change and therefore the ARI was calculated.

Mean baseline MAP value was 67.6±0.36mmHg. A scatter plot of synchronic CFI over MAP data was outlined to demonstrate the AR curve (Fig 5 (right)), with a cutoff MAP distinguishing between a plateau and a linear behavior obtained in two distinct MAP ranges (only the periods of significant MAP change are plotted). From this figure it is evident that cutoff MAP for this animal was observed at 100mmHg. To validate the MAP cutoff, the slope of CFI change in response to MAP change was calculated for both MAP regions, demonstrating significantly different slope values for MAP under 100 mmHg (slope = 0.108, 95% confidence interval [0.104, 0.112]) and over 100 mmHg (slope = 0.556, 95% confidence interval [0.544, 0.568]). We therefore defined this point as the Upper Limit of Autoregulation (ULA).

The lower limit of autoregulation (LLA) could not be determined within the obtained MAP range, as no additional slope was identified.

**Fig 6:**
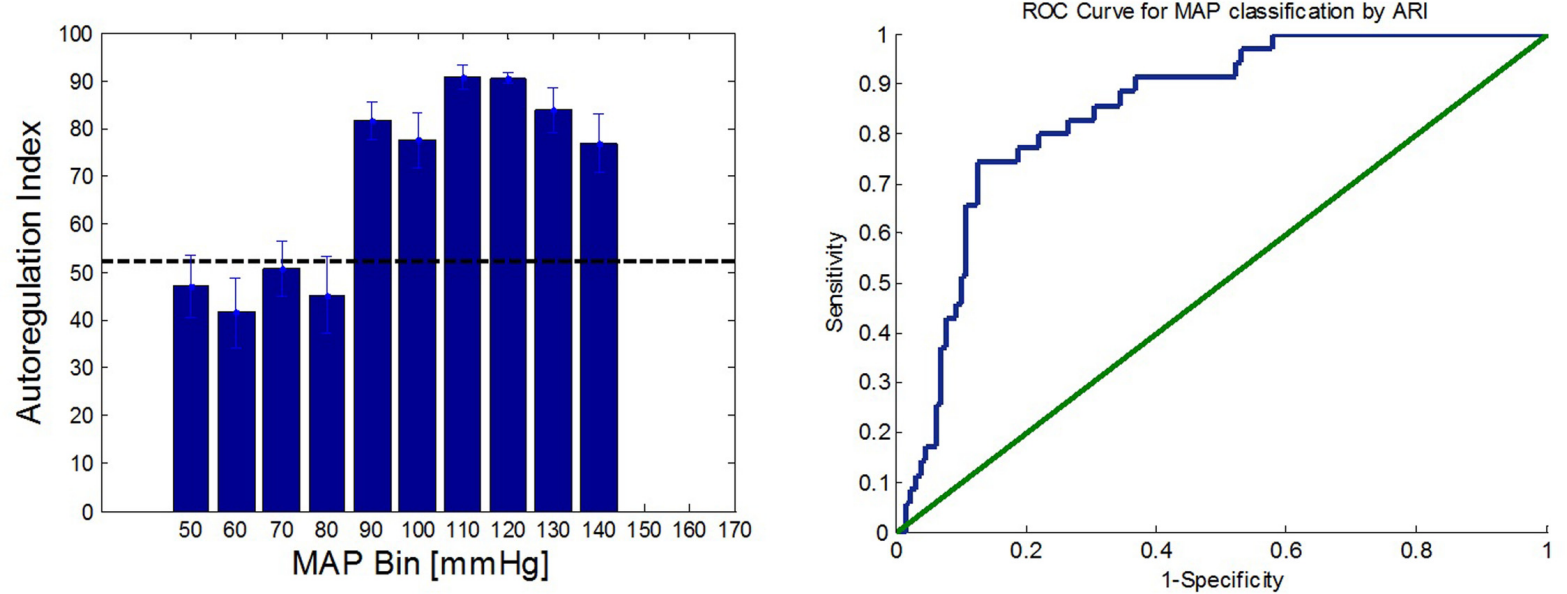
Left–Bar Diagram of averaged autoregulation index for each 10mmHg MAP segment. Error bars represent the standard error of the mean. Threshold value is 52. Right–ROC analysis for MAP classification as over or under the ULA. Area Under the Curve (AUC) is 0.848 (95% confidence interval [0.783, 0.912]).

To further exemplify the ARI ability to differ between MAP values over and under the ULA, ARI bar graph and corresponding ROC analysis are presented in Fig 6.

The bar height chart represents the mean ARI obtained in each MAP segment (error bars stand for the standard error of the mean in each segment). ARI values obtained for MAP over the ULA were significantly higher than those obtained for MAP under the ULA (unpaired t-test, p<0.001). ARI threshold used was 52 (Fig 6 Left).

The ROC analysis for classifying MAP as over or under the ULA according to its matching ARI yielded an Area Under the Curve (AUC) of 0.848 (95% confidence interval [0.783, 0.912]), indicating a good accuracy in MAP discrimination.

## Discussion

The development of a novel device incorporating MAP and CFI measurements to enable real time autoregulation monitoring is presented. Its ability to correlate the two parameters in real time and provide an index indicating on autoregulation is introduced and validated in both *in-vitro* experiments and a preclinical case study. Results suggest that using such a tool, autoregulation boundaries as well as its impairment or functioning can be identified and assessed.

The combination of CBF monitoring along with synchronized systemic MAP was extensively researched and was suggested to be beneficial in providing an indication of the autoregulatory state[35]. In those studies, CBF was not directly measured, instead, surrogate parameters, such as TCD cerebral blood flow velocity (CBFV), ICP and NIRS oximetry, were used. The use of these parameters has substantial limitations. For TCD, which insonates large vessels, the flow measurement requires the assumption of constant vessel diameter, an inherent problem when assessing the vasorectivity based mechanism of AR. In addition, TCD is difficult to use continuously, especially in an environment of electrical noise, such as the operating room. Because of the difficulties in acquiring and maintaining a stable TCD signal, its mean velocity index (Mx) is not the most widely used monitor for assessing autoregulation, although it was arguably the first[10]. Another widely studied technique involves the correlation of MAP with intracranial pressure (ICP) yielding a pressure reactivity index (PRx), which is also an indirect indication for the patient’s autoregulatory state[9–11]. This method is based on pressure AR theory rather than on CBF AR. But more important, it is invasive and therefore can be applied to a limited patient population, namely severe traumatic brain injury who are subject to intensive intracranial monitoring.

Regional NIRS oximetry (rStO2), which measures tissue mixed venous blood oxygen saturation, assumes that changes in blood flow have direct implication on rStO2. Yet this parameter is proportional to hemoglobin concentration in the tissue, which does not necessarily correspond to blood flow. An additional disadvantage of this technology is the influence of superficial extracerebral layers on the signal which has been shown to be significant in a number of studies[36, 37]. This may introduce misleading conclusions while assessing AR and pursuing the difference between systemic and cerebral behaviors.

Ornim’s Ultrasound Tagged Near-Infra Red (UT-NIR) based device (the c-FLOW and its predecessor the CerOx) directly monitors the CBF trend continuously and non-invasively. Preliminary studies which utilize its cerebral flow index (CFI) for autoregulation assessment were conducted[28, 29] and showed good agreement between CBF autoregulation monitored by CerOx (correlation flow index (CFx)) compared with the TCD-based monitoring (mean velocity index (Mx))[28]. Therefore, the development of a new monitor using the linear correlation construct is a logical choice. This device which measures both MAP and CFI data and correlates them in real-time to obtain an indication for the autoregulatory state presented here, opens a new opportunity for an integrated monitor.

Autoregulation assessment in this study is based on the relationship between CBF (as expressed by CFI) to MAP changes. By definition, AR refers to CBF response to MAP changes and the ability of the brain to maintain constant CBF in a certain range of MAP values. Hence, assessment of the relationship between synchronized MAP and CBF signals should provide a solid indication for the patient’s autoregulatory state. It is well established that there are additional factors which may contribute to the estimation of AR condition, such as oxygen saturation[15] and blood volume[38]. Future work should investigate the possibility to combine these three parameters, using the UT-NIR technology of the c-FLOW, as suggested by Brady[10], to assist in drawing a bigger picture as to the patient’s autoregulation condition. Yet, even the narrower observation on MAP and CFI only described here, might be sufficient, at first, and provide valuable information with straightforward clinical virtues.

Experiment in laboratorial environment was designed to validate the device ability to measure both signals simultaneously and correlate them in real-time. The pressure wave produced by the experimental setup (illustrated in Fig 2B) although not being identical to a typical BP waveform, modeled it adequately. BP parameters could be continuously calculated by both the c-FLOW and other vital sign monitors (such as Phillips intellivue MP50), indicating that it can serve as a reliable BP simulator for multiple purposes. Proper acousto-optic modeling of CBF using the described phantom was previously proved and validated[30, 31]. Thus, both MAP and CFI trends were reliably modeled.

Though modeling CBF adequately, it is important to notice that the acousto-optic flow phantom utilized in the experiment is not ideal. Small instantaneous changes in flow within its channels may occur due to the syringe pump accuracy in flow supply or the uneven dispersion of scatterer centers within the flowing fluid. This factors may contribute to the relatively high CFI coefficient of variance obtained for the stable flow in sensor 2 (CV = 0.2).

Obtained results confirmed the algorithm ability to distinguish cases in which both MAP and CFI trends are changing concurrently, and differ them from cases in which CFI does not track MAP changes using the cross correlation based ARI (Fig 5). Theoretically, ARI values range between 0–100, with 0 representing no correlation and 100 representing a perfect one. In our *in-vitro* experiment, ARI values of 37.66±17.99 and 61.74±21.36 were associated with AR and no AR (NAR) conditions, respectively. These absolute obtained values are not compatible with the hypothetical values of 0 versus 100, however, the two groups were significantly different (P<0.001). Further work should be dedicated to noise reduction and expansion of the obtained ARI values range to improve and enhance its ability to differ between the AR conditions. Based on previous studies to track AR utilizing correlation between MAP and CBF[13, 14, 25, 28, 29, 39], the above results support the use of the calculated ARIs for autoregulation monitoring.

The experimental setup described here, combining both BP and flow modeling, is modularly built enabling simultaneous control on each of its parameters independently. Thus, in future studies it may be used to simulate more complicated protocols, different disease states, and various relationships between CBF and MAP. It may be utilized to further investigate the algorithm and predict its response to different conditions such as CBF change without changes of MAP (as can happen in severe head injury[40]) or delayed response of CBF to a MAP change (as in hypothermia cases when CBF becomes uncoupled from the metabolic rate[41] or in case of delay in autoregulatory action depending on PaCO_2_ [2]), etc.

Additional validation for the c-FLOW-AR ability to detect AR-related trends was provided in the swine case study. The upper limit of autoregulation was clearly identified by both slope analysis of CFI versus MAP scatter plot (Fig 5(right)), and using the calculated ARI which quantitatively differed MAP segments under and over the ULA (Fig 6).

For this piglet, ULA was identified at a MAP of 100mmHg. This value may seem rather low comparing to the traditional autoregulation curve described in humans, ranging between 50mmHg to 150mmHg, however in previous studies performed with pigs similar values were reported[42].

Though ULA was distinctly evident in the presented case study, the LLA was not identified within the obtained MAP range. According to Fig 5, the lowest measured MAP was approximately 40mmHg. For this swine, lower MAP values weren’t attainable even while increasing Nitroprusside to a dose of 62ml/hr. In a study designed to describe changes of LLA according to temperature[43], LLA in normothermia was achieved at 38±8mmHg with LLA values of even lower than 35mmHg. We therefore suggest that the LLA might be lower than the lowest obtained BP in our study, hence it was not apparent. Further studies, using higher drug doses or different protocols, should be carried to identify LLA as well.

Beyond visual examination and slope analysis for the CFI versus MAP scatter plot given in Fig 5(right), a quantitative evaluation was performed using the calculated autoregulation indexes. Observation on the binned averaged ARI in Fig 6, unambiguously and quantitatively differ the two mentioned MAP segments (under and over the ULA). ARI threshold value was selected to be 52, which is slightly higher than the values reported in previous studies (0.4–0.45, which correspond to 40–45 in our ARI units) [13, 14, 28]. Most studies were carried using CBF velocity as a surrogate to CBF rather than the measurement of CFI, thus are not necessarily comparable in terms of absolute correlation values and thresholds. Yet, needless to say that in order to establish a reliable and repetitive threshold value for CFI and MAP correlaion index, more statistics should be obtained.

The preclinical case study presented here provides a good evidence for the ability of the c-FLOW-AR to provide real-time autoregulation index *in-vivo*. This is only a preliminary validation of this device performance and recurrent studies should provide more statistics. As several other studies[28, 29, 39] were already conducted to evaluate autoregulation using UT-NIR technology for CBF monitoring with the difference of having an independent MAP tracking, similar results are expected using the integrated device.

The c-FLOW-AR has a great potential in outlining autoregulation and becoming useful in clinical practice for individualized management of BP, and improving patients outcome. Studies in cardio vascular surgeries suggested that it might be able to predict postoperative complications, such as acute kidney injury[39] or delirium[44]. They further indicated that individualizing MAP management during Cardio Pulmonary Bypass surgery based on CBF autoregulation monitoring holds promise as an innovative approach to ensure organ perfusion and reduce the risk of postoperative complications after cardiac surgery.
